# Unravelling the Impact of Foreign Bodies on Bronchiectasis: Insights From a Case Series Study

**DOI:** 10.7759/cureus.69475

**Published:** 2024-09-15

**Authors:** Prasanna Kumar T, Shashidhar S Vananjakar, Tanisha Saleem, Pragati Rao D, Sruthy Vijayan

**Affiliations:** 1 Respiratory Medicine, M S Ramaiah Medical College and Hospital, Bangalore, IND

**Keywords:** children vs adults, flexible fiberoptic bronchoscopy (ffb), hrct thorax, pin, pleural empyema, segmental bronchiectasis, swallowed foreign body, vegetative material

## Abstract

Foreign body (FB) aspiration in adults is a rare yet critical event that can mimic chronic respiratory conditions such as asthma or bronchitis, often causing delays in diagnosis and treatment. This case series explores the presentations of four adult patients, each with a prolonged history of chronic cough, who were later discovered to have aspirated foreign bodies. Initial misdiagnoses and the limitations of high-resolution CT scans in detecting these foreign bodies contributed to delays in reaching an accurate diagnosis. Interestingly, three of the cases involved aspirated vegetative matter, which went undetected on imaging and was only identified through bronchoscopy. The fourth patient, a young adult male, had aspirated a safety pin, which led to empyema, a severe complication highlighting the risks associated with delayed diagnosis. A key finding in this series is the significant role of flexible bronchoscopy in both diagnosing and managing FB aspiration. In each case, flexible bronchoscopy, guided through a rigid bronchoscope, was instrumental in successfully removing the foreign bodies, even in complex cases involving vegetative material or sharp objects. This case series underscores the importance of considering FB aspiration in adults with unexplained chronic cough, especially when conventional imaging does not reveal a clear cause. The diagnosis of airway foreign body requires a thorough clinical history and assessment of risk factors, with bronchoscopy serving as a crucial diagnostic and therapeutic tool when CT scans are inconclusive and stressing the need for timely diagnosis and intervention to prevent severe complications and improve patient outcomes.

## Introduction

Bronchiectasis resulting from foreign body (FB) aspiration comprises a small number of cases. Foreign body aspiration is a rare but life-threatening event. Bronchial foreign bodies are uncommon in adults. Significant neurological impairment, alcohol and drug intoxication, and poor dentition are usually identified as risks associated with bronchial foreign bodies in adults [1]. Diagnosing foreign body aspiration as a cause of bronchiectasis, while relatively rare, is essential, as many patients initially present with chronic cough and may be erroneously treated for conditions like asthma or chronic bronchitis. Consequently, prioritizing imaging modalities, particularly in cases of segmental bronchiectasis, is imperative for accurate diagnosis and timely intervention. History is the most important factor in diagnosis. Sometimes, aspirated tracheobronchial foreign bodies have been treated without diagnosis or with incorrect diagnoses, such as chronic pneumonia, bronchitis, asthma, bronchiectasis, and even tumors, for many years [2]. Bronchoscopy remains the gold standard for diagnosing and treating foreign body aspiration from the lower respiratory tract.

## Case presentation

**Figure 1:**
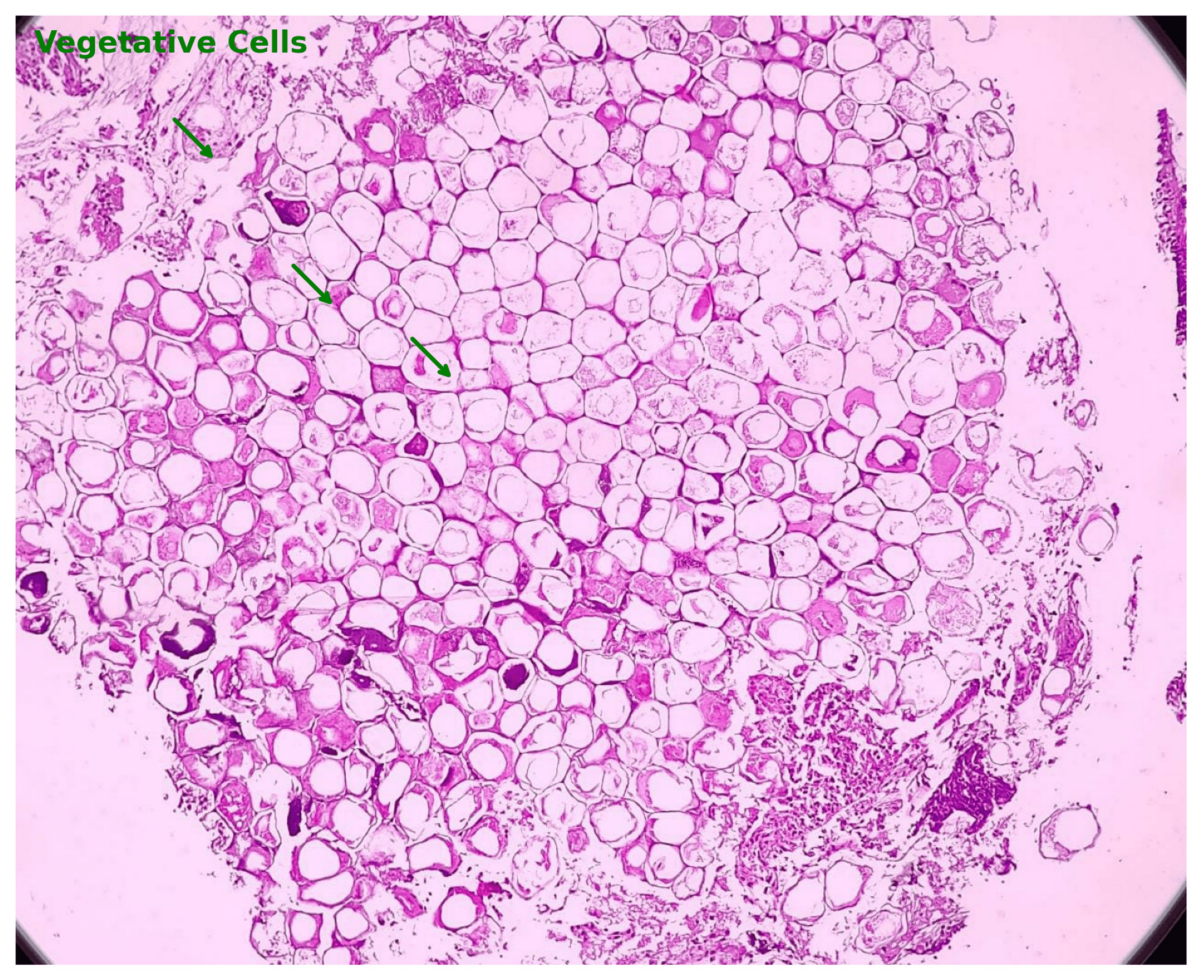
Histopathological section of foreign body showing vegetative cells Case 1: This figure displays a histopathological section with vegetative cells prominently marked by green arrows. These cells are evident as larger, polygonal structures, likely remnants of plant material, within the tissue section.

**Figure 2:**
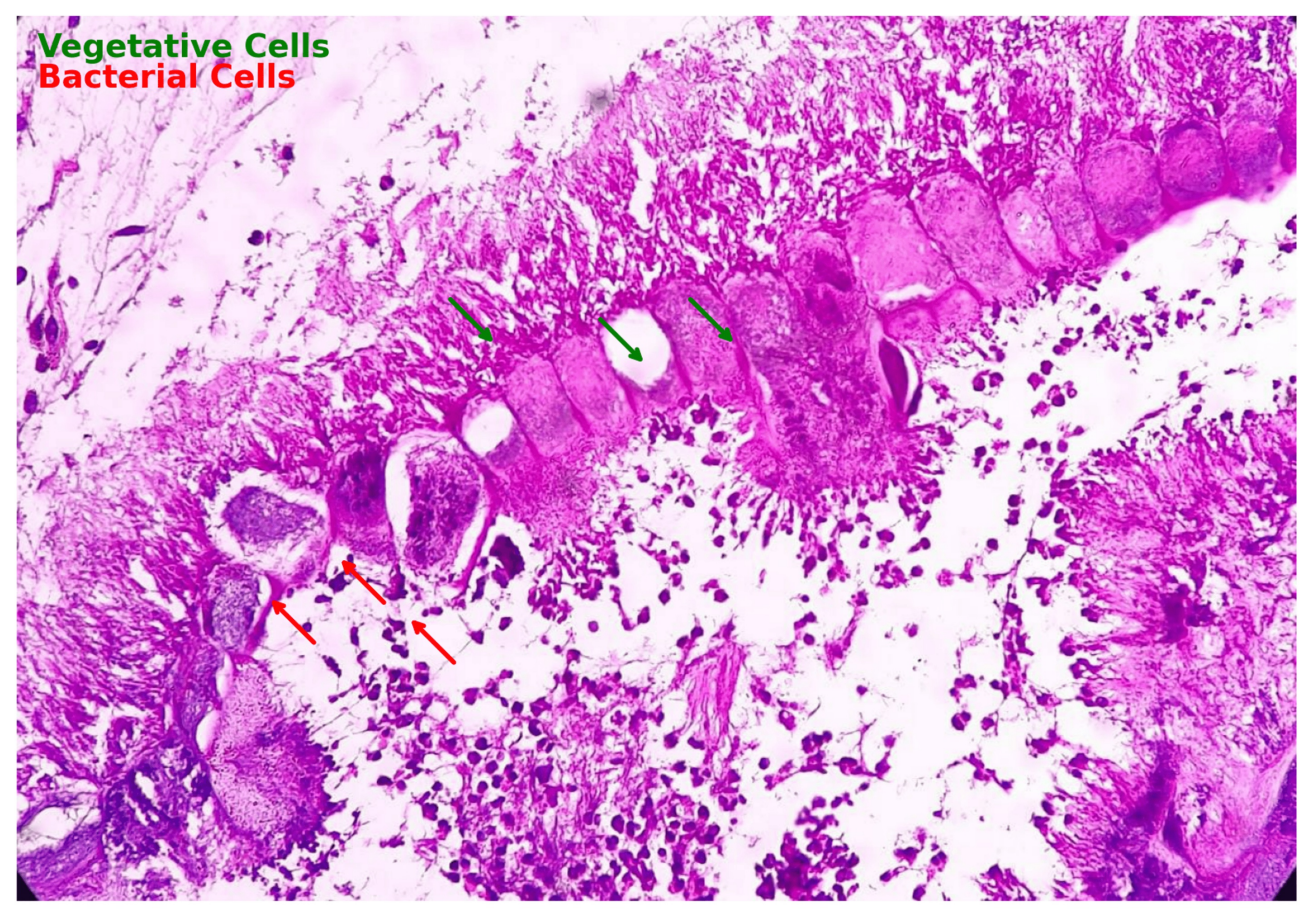
Histopathological section of foreign body showing vegetative and bacterial cells Case 2: This figure illustrates a histopathological section where bacterial cells (marked by red arrows) and vegetative cells (marked by green arrows) are identified. The bacterial cells appear as small, dot-like structures scattered throughout the tissue, while the vegetative cells are larger, irregular fragments indicative of organic material.

Four adult cases, all aged between 18 and 86 years and displaying varying durations and types of symptoms, presented with chronic productive cough, without any prior history of allergic reactions or respiratory illnesses (Table 1). Imaging studies, including chest X-ray and high-resolution computed tomography (HRCT) of the thorax, identified segmental bronchiectasis in the right lower lobes in three cases, and one case also exhibited empyema in the left lung. Initially, no mucus plugging, or foreign bodies were reported in these three cases. However, upon conducting diagnostic bronchoscopy, unexpected FBs were found in three of the cases, with biopsies revealing vegetative matter in two of them. A safety pin was also discovered in one of the cases. The foreign bodies were successfully removed using a fiber optic bronchoscope, facilitated by rigid bronchoscopy, employing alligator forceps and a flexible cryoprobe with an outer diameter of 1.7 mm. An interesting aspect was that three individuals had biopsy-proven vegetative matter causing chronic symptoms, which were initially managed in local hospitals. The duration of symptoms in the first case was noted as two months, reflecting the chronic nature of the condition (Figures 1, 2).

**Table 1:** Clinical Presentation, Imaging Findings, and Bronchoscopy Outcomes in Four Cases of Chronic Foreign Body Aspiration HRCT: high-resolution computed tomography

Sl.No	Age/Sex	Clinical presentation	Duration	HRCT thorax findings	Bronchoscopy findings
Case 1	84Y/F	Recurrent episodes of cough with expectoration and fever	2 months	Volume loss with cystic bronchiectasis of left lower lobe. (Figure 3)	Whitish endobronchial mass noted in posterior segment of left lower lobe with mucoid secretions (Figure 4)
Case 2	86Y/F	Easy fatiguability & cough with expectoration	15 days	Patchy consolidation with cystic bronchiectasis in posterior and medial basal segments of right lower lobe. (Figure 5)	Right- ectatic segments with right lower lobe whitish endobronchial mass? Left -lingular segment narrowed with endobronchial mucosal oedema (Figure 6)
Case 3	58Y/F	Cough with expectoration, breathlessness & loss of weight	3-4 months	Cylindrical bronchiectasis & nodularity of the right lower lobe. (Figure 7)	Endobronchial variations noted & mucus noted in right lower lobe segment. With suction? foreign body. (Figure 8)
Case 4	18Y/M	On & off cough with minimal expectoration	2 months	Radio-opaque long foreign body? Pin in left lower lobe bronchus, collapse consolidation led to loculated effusion in posterior aspect of left thorax. (Figure 9)	Purulent secretions oozing in the left lower division bronchus. Foreign body visualised in the apical segment; removal attempted by alligator forceps. (Figure 10)

**Figure 3:**
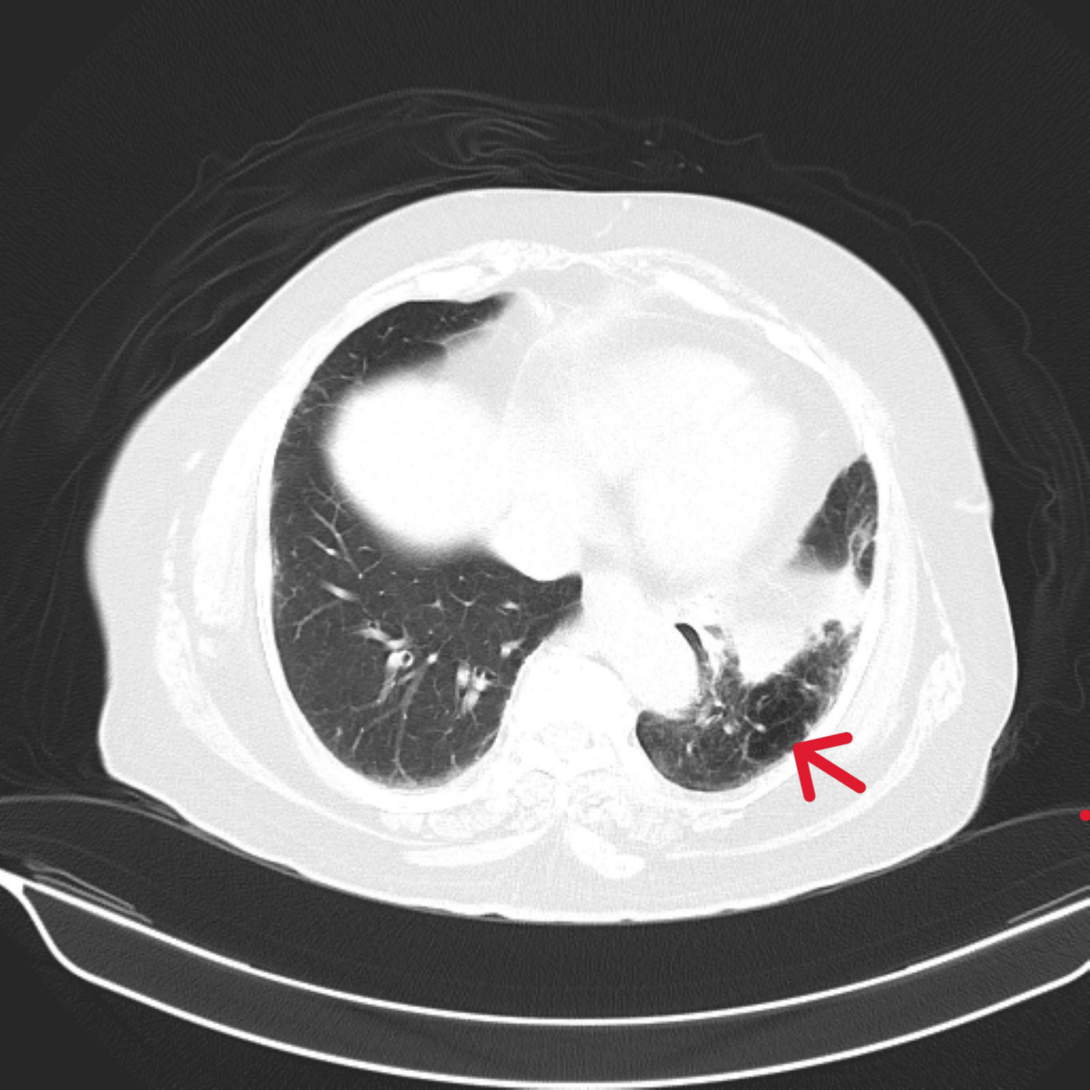
Axial section of a computed tomography (CT) scan of the chest Case 1: Volume loss with cystic bronchiectasis of left lower lobe (red arrow).

**Figure 4:**
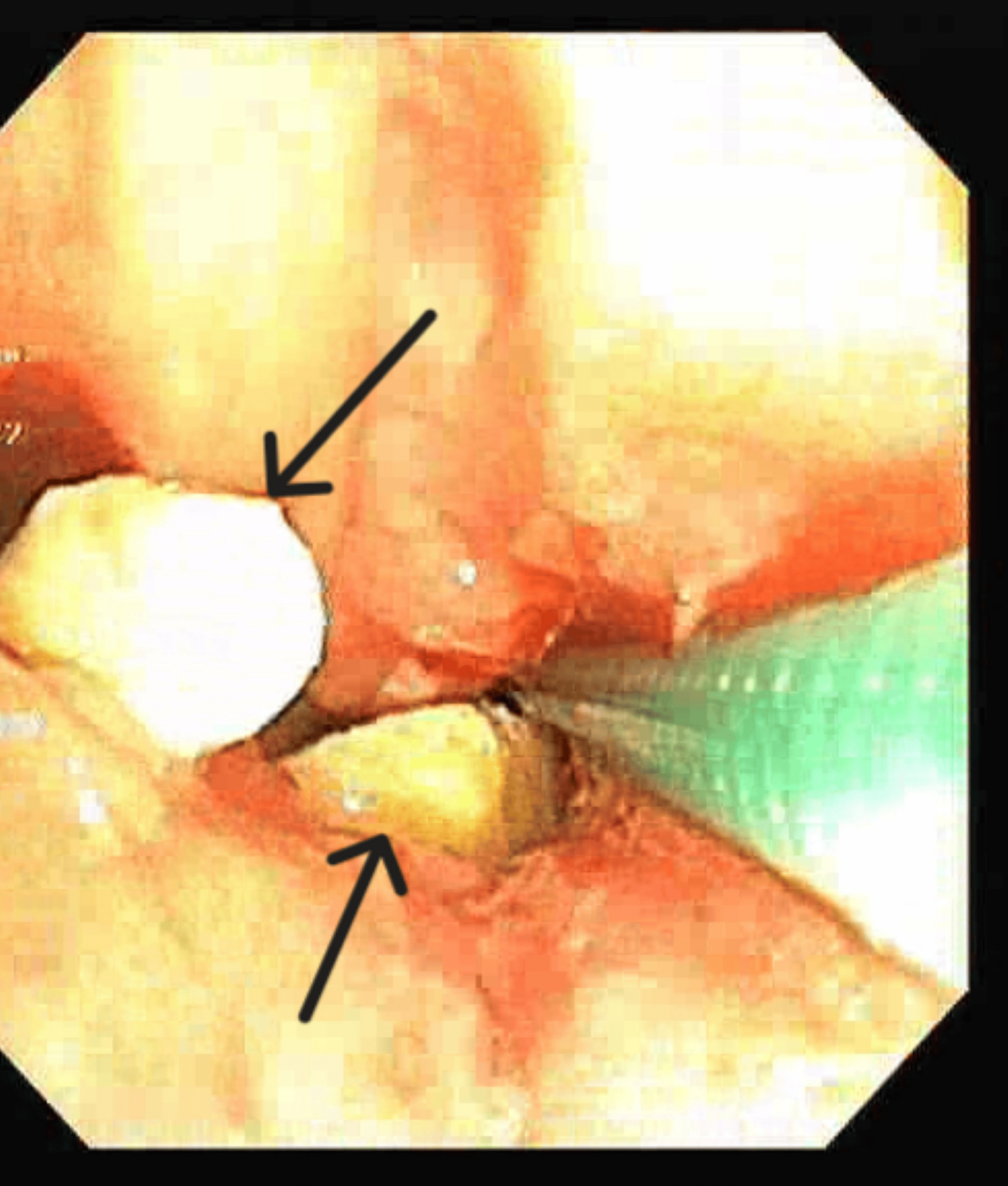
Bronchoscopic image showing a foreign body in the left lower lobe. Case 1: Endobronchial fragmented foreign body (black arrows) located in the left lower lobe segment.

**Figure 5:**
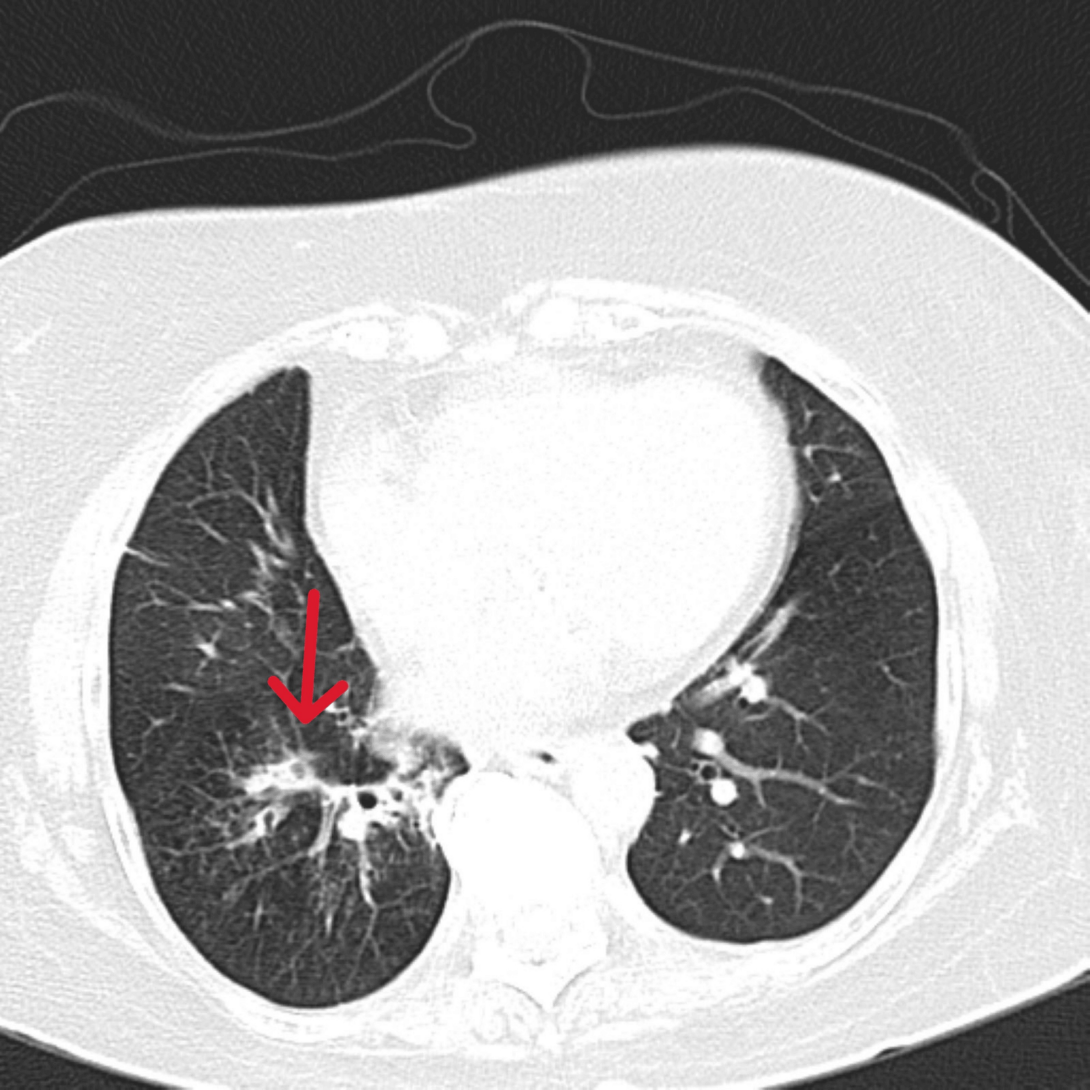
Axial section of a CT Chest Case 2: Patchy consolidation (red arrow) with cystic bronchiectasis in posterior and medial basal segments of right lower lobe.

**Figure 6:**
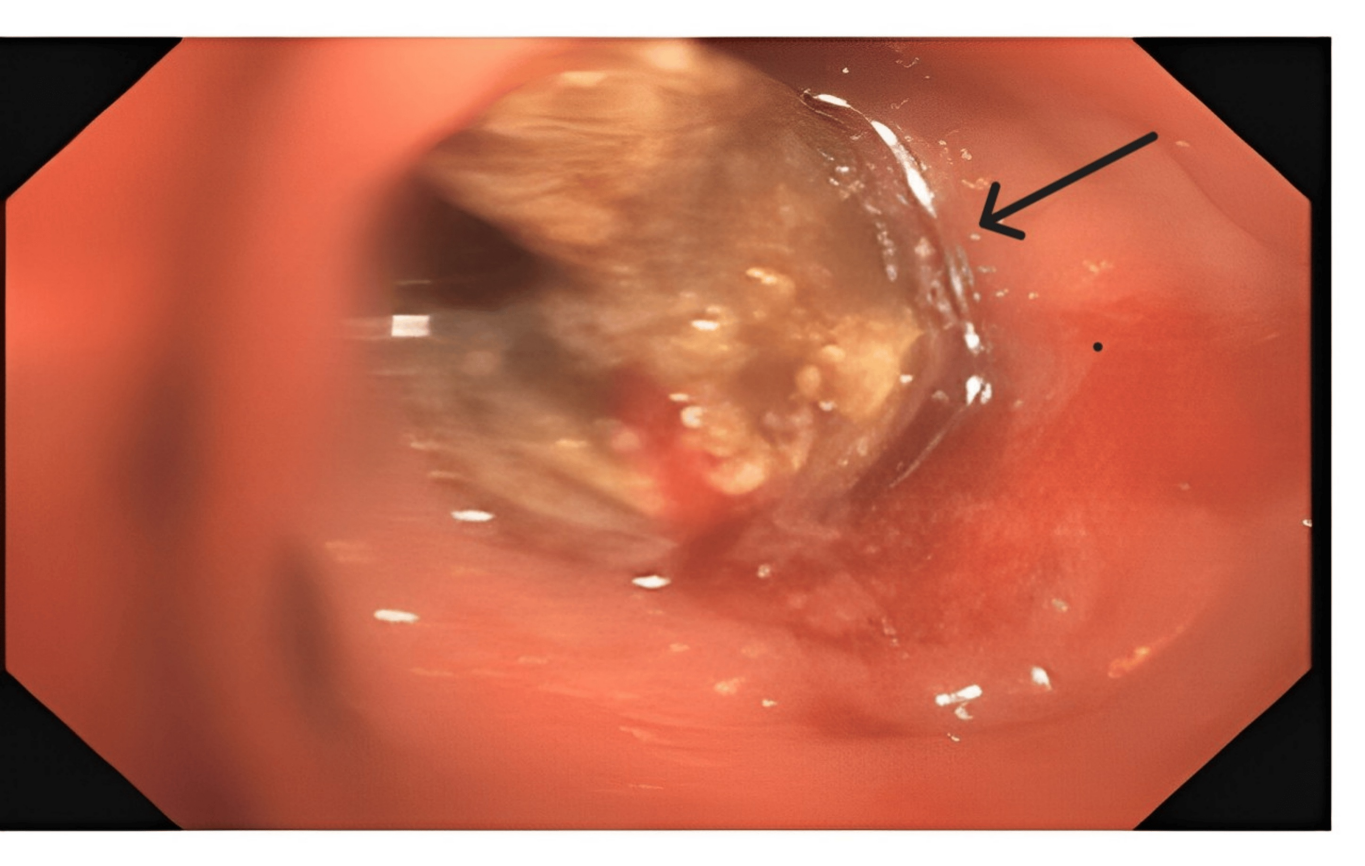
Bronchoscopic image showing a foreign body in the right lower lobe. Case 2: Endobronchial mass? foreign body in right lower lobe with oedematous mucosa (black arrow).

**Figure 7:**
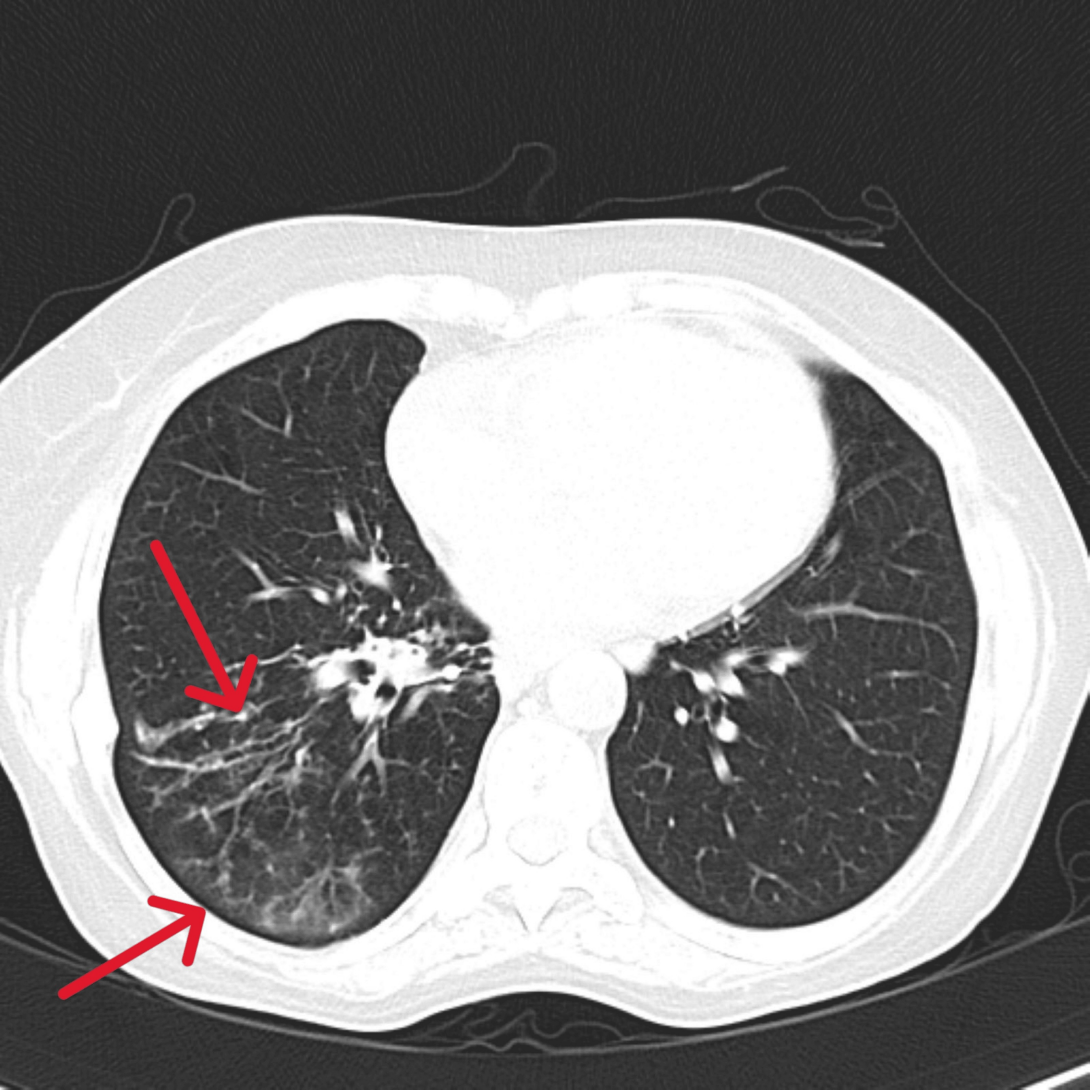
Axial section of a CT Chest. Case 3: Cylindrical bronchiectasis (red arrow) and nodularity of the right lower lobe, with ground glassing at the periphery.

**Figure 8:**
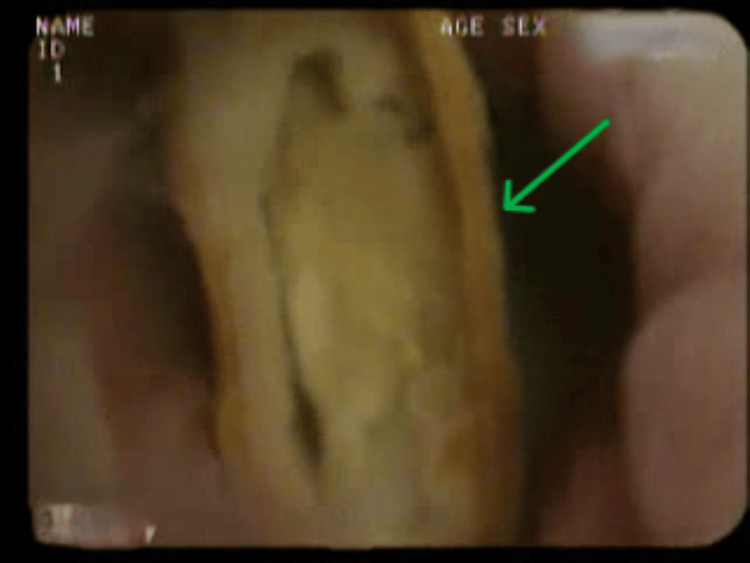
Bronchoscopic image showing a foreign body in the right lower lobe. Case 3: An endobronchial foreign body resembling seed (green arrow) right lower lobe.

**Figure 9:**
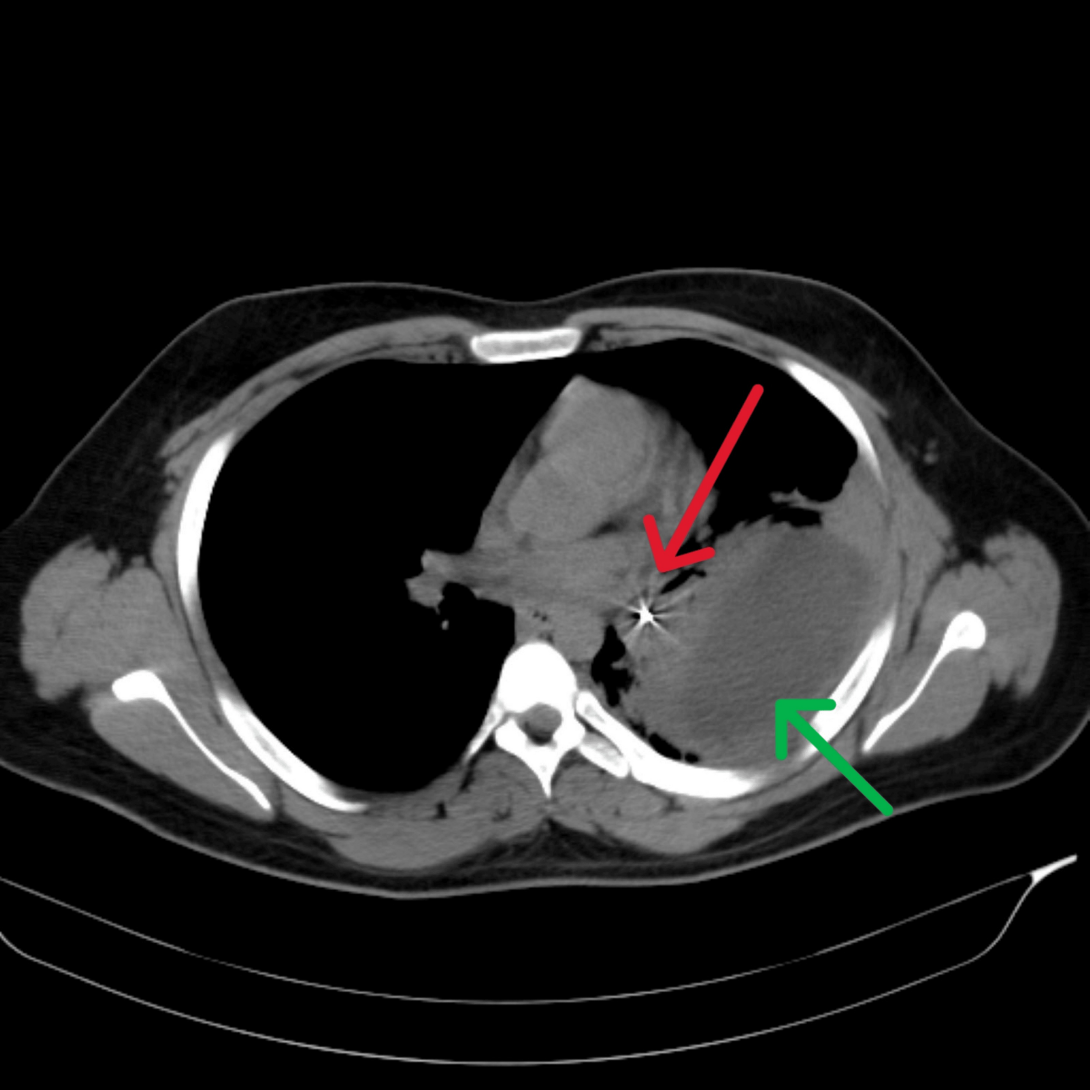
Axial section of a CT Chest. Case 4: A radio-opaque, long foreign body (pin) is seen in the left lower lobe bronchus (red arrow), causing collapse consolidation and resulting in a loculated effusion in the posterior aspect of the left thorax (green arrow).

**Figure 10:**
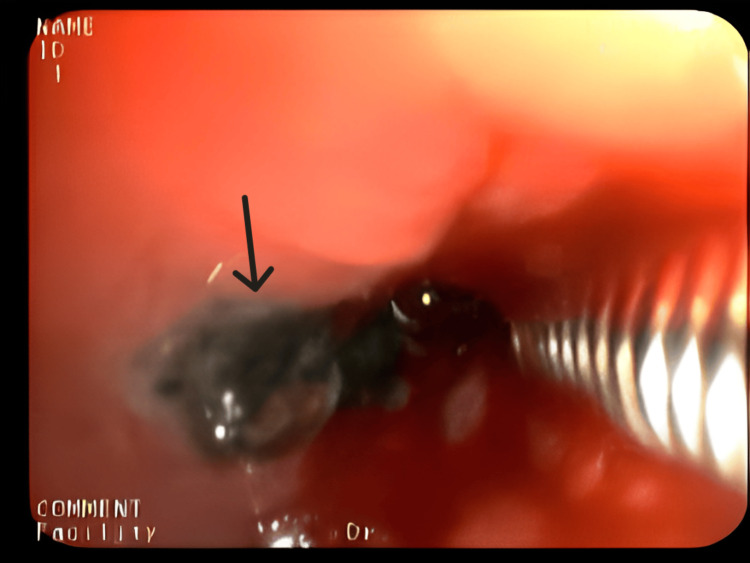
Bronchoscopic image showing a foreign body in the left lower lobe. Case 4: Black granulation tissue surrounding the foreign body (pin) in the left lower lobe segments, with edema of the surrounding tissue (black arrow).

Case 1 was an 84-year-old female presented with recurrent episodes of cough with expectoration and fever persisting for two months. Chest X-ray (CXR) showed no evidence of parenchymal disease. Sputum analysis was inconclusive. Blood investigations revealed leucocytosis, predominantly neutrophilic. She was subjected to HRCT imaging, which showed volume loss with cystic bronchiectasis in the left lower lobe (Figure 3). Diagnostic bronchoscopy revealed a whitish endobronchial mass in the posterior segment of the left lower lobe with mucoid secretions (Figure 4). Upon further exploration, a foreign body was detected, later identified as vegetative matter. She was started on broad-spectrum oral antibiotics and followed up in the outpatient department (OPD).

Case 2 was an 86-year-old female who reported symptoms of easy fatigability and cough with expectoration for 15 days. CXR showed inhomogeneous opacity in the right lower zone, paracardiac region. Sputum analysis was inconclusive. HRCT findings revealed patchy consolidation with cystic bronchiectasis in the posterior and medial basal segments of the right lower lobe (Figure 5). Bronchoscopy findings showed a whitish endobronchial mass in the ectatic segments of the right lower lobe and mucosal edema in the left lingular segment (Figure 6). Similar to the first case, vegetative matter was discovered upon biopsy and was determined to be the cause of the chronic symptoms. The patient was discharged with oral antibiotics and bronchodilator nebulizations and followed up; later, the patient experienced fewer exacerbations.

Case 3 was a 58-year-old female who experienced a persistent cough with expectoration, breathlessness, and weight loss over three to four months. Initial CXR was normal with no evidence of pneumonia, and blood investigations were within normal limits. HRCT imaging revealed cylindrical bronchiectasis and nodularity of the right lower lobe (Figure 7). Bronchoscopy showed variations in the endobronchial architecture and the presence of mucus in the right lower lobe segment. Further suctioning suggested the presence of a foreign body, which was confirmed as vegetative matter upon biopsy (Figure 8). She was treated with oral broad-spectrum antibiotics and bronchodilator nebulizations.

Case 4 was an 18-year-old male, the youngest in the group, presented with a two-month history of intermittent cough and minimal expectoration. CXR revealed left pleural effusion with a radio-opaque lesion in the left lower zone, raising suspicion of a foreign body. Complete blood count (CBC) showed leukocytosis. The patient was initiated on parenteral antibiotics and supportive care. HRCT imaging identified a long, radio-opaque foreign body, suspected to be a safety pin, lodged in the left lower lobe bronchus, resulting in collapse, consolidation, and loculated effusion in the posterior aspect of the left thorax (Figure 9). Bronchoscopy revealed purulent secretions from the left lower division bronchus and a foreign body in the apical segment. Attempts at removal using alligator forceps were unsuccessful (Figure 10), and the patient underwent removal via rigid bronchoscopy at another facility. Empyema was managed with pigtail drainage and continued antibiotic therapy.

## Discussion

This case series documents four adult patients initially misdiagnosed with airway obstruction diseases, treated for two to three years before FB aspiration was identified. The study emphasizes the critical importance of timely and accurate diagnosis, especially in cases of chronic cough and respiratory symptoms. Three cases involved biopsy-proven vegetative matter (Figures 1, 2), which caused chronic symptoms and was missed on initial imaging but discovered during flexible bronchoscopy. Imaging, including CT scans (Figures 3, 5, 7, 9), revealed segmental bronchiectasis in these cases, but CT thorax scans failed to detect the organic foreign bodies due to limitations in identifying radiolucent objects, especially those composed of vegetable matter. A young adult male who aspirated a safety pin developed empyema; a complication detected through imaging. Foreign body aspiration in adults is usually seen in the very elderly or in individuals with underlying neurological conditions, psychiatric illnesses, Alzheimer’s disease, or head trauma [3]. In a retrospective history of the three cases, it was noted that they had experienced a choking sensation upon eating in the past, with the longest occurrence being four years ago. Bronchoscopy ultimately enabled the detection and safe removal of the foreign bodies in all cases, highlighting its critical role in diagnosis and treatment.

The importance of bronchoscopy, CT imaging, and X-rays in detecting foreign bodies cannot be overstated. While CT scans are generally more sensitive than X-rays, particularly in detecting FB-associated complications like abscesses or empyema, they may fail to identify certain radiolucent objects, especially organic materials like vegetative matter [4]. This was evident in three of the four cases, where CT scans did not reveal the foreign objects, but bronchoscopy provided definitive detection. The opacity of foreign objects and the density of surrounding tissues affect the visibility on both CT and X-rays, with faintly opaque objects near osseous structures posing additional challenges. In cases of radiolucent foreign bodies, bronchoscopy becomes indispensable, offering a direct view into the airways and enabling the removal of the FBs. 

This study underscores the need for bronchoscopy as a first-line diagnostic tool when chronic respiratory symptoms persist, especially when imaging is inconclusive. Flexible bronchoscopy not only diagnosed the foreign bodies in these cases but also guided subsequent therapeutic decisions (Figures 4, 6, 8, 10). While CT imaging is helpful in identifying associated complications, such as empyema or fistula formation, and the use of intravenous contrast can enhance its diagnostic value for inflammatory lesions [5], bronchoscopy proved vital for detecting organic FBs missed on imaging. X-rays, although useful, have limitations in detecting faint or radiolucent objects and are less sensitive than CT scans and bronchoscopy.

The findings underscore the necessity of using a combination of imaging techniques, such as X-rays and CT scans, in conjunction with bronchoscopy for a thorough evaluation of suspected FB aspiration. In our case series, FBs were detected during bronchoscopy. Once identified as vegetative matter, we proceeded with a subsequent procedure using rigid bronchoscopy as the conduit for removal.

**Figure 11:**
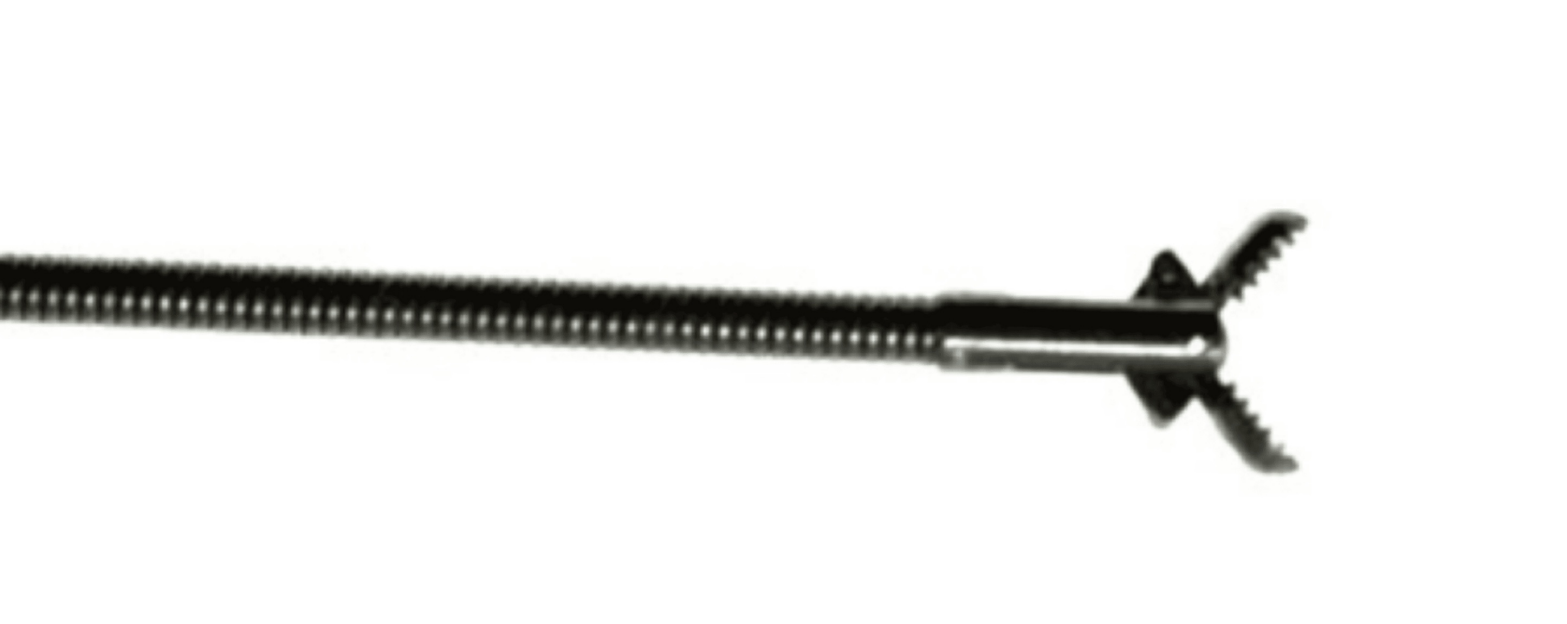
Alligator Forceps

**Figure 12:**
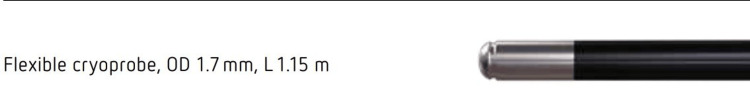
Flexible Cryoprobe outer diameter (OD) 1.7mm, L 1.15 m

Initially, alligator forceps (Figure 11) were used to attempt the removal of the FBs. However, due to their delicate and fragmented nature, multiple attempts were required. In cases where the FBs were particularly fragile or organic, a flexible cryoprobe (outer diameter (OD) 1.7 mm) was used in the same setting as an adjunct to the rigid bronchoscopy (Figure 12). The cryoprobe proved particularly effective for managing softer and more fragile materials, facilitating successful removal. Other methods for FB removal, such as gaskets and specialized retrieval devices, may also be considered depending on the type and location of the FB. These techniques can provide additional options for effective retrieval when conventional methods are insufficient

The series also contributes to the limited literature on adult FB aspiration in India, stressing the need for increased awareness and prompt intervention to prevent complications and improve outcomes. 

Similar studies in the literature have also reported findings consistent with those described in this case series. For instance, Chen et al. (1997) identified food-related foreign bodies, such as bone fragments and seeds, in adult patients, highlighting the challenge of detecting organic materials on imaging and the crucial role of bronchoscopy in their removal. Wang et al. (2016) described a case where Chinese herbal medicine, aspirated and undetected for a decade, was only discovered through bronchoscopy, further underscoring the limitations of CT scans in detecting radiolucent objects like vegetative matter. Additionally, Kuba et al. (2015) and Metin et al. (2016) reported cases of unusual and chronic foreign body aspiration, where bronchoscopy was critical in identifying objects like parts of a pen and food particles, both of which were missed by conventional imaging. Pellissier et al. (2017) documented a case where a bottle cap lodged in the bronchus for 41 years was finally discovered via bronchoscopy, despite being missed on earlier radiographs and CT scans. These studies collectively emphasize the essential role of bronchoscopy in diagnosing and managing foreign body aspiration, especially when imaging modalities like CT and X-rays fall short in detecting organic or radiolucent materials [6-10].

## Conclusions

The study provides crucial insights into the complexities of managing FB aspiration in adults, analyzing four cases to reveal challenges in diagnosis and treatment, while demonstrating the effectiveness of rigid bronchoscopy for FB removal. The fragile nature of vegetable matter FBs makes them difficult to extract, but the cryoprobe efficiently addresses this issue, ensuring safe removal. For harder objects, such as metallic FBs, alligator forceps used with flexible bronchoscopy, with rigid bronchoscopy as a conduit, proved highly effective. A case involving a safety pin FB complicated by empyema underscores the dangers of delayed diagnosis, yet successful removal through rigid bronchoscopy emphasizes the importance of prompt action to prevent complications. Overall, the study stresses a multidisciplinary approach and further research to enhance FB aspiration management and patient outcomes.
